# Nanosilver-based materials as feed additives: Evaluation of their transformations along *in vitro* gastrointestinal digestion in pigs and chickens by using an ICP-MS based analytical platform

**DOI:** 10.1007/s00216-024-05323-8

**Published:** 2024-05-22

**Authors:** Khaoula Ben-Jeddou, Mariam Bakir, María S. Jiménez, María T. Gómez, Isabel Abad-Álvaro, Francisco Laborda

**Affiliations:** Group of Analytical Spectroscopy and Sensors (GEAS), Institute of Environmental Sciences (IUCA), University of Zaragoza, Pedro Cerbuna 12, 50009 Saragossa, Spain

**Keywords:** *In vitro* gastrointestinal digestion, Feed additive, Silver based nanomaterial, ICP-MS, SP-ICP-MS, HDC-ICP-MS

## Abstract

The use of a new nanomaterial in the feed chain requires a risk assessment that involves in vitro gastrointestinal digestions to predict its degradation and oral exposure to nanoparticles. In this study, a nanosilver-based material was incorporated into pig and chicken feed as a growth-promoting additive and subjected to the corresponding in vitro gastrointestinal digestions. An inductively coupled plasma mass spectroscopy (ICP-MS) analytical platform was used to obtain information about the silver released in the different digestion phases. It included conventional ICP-MS for total silver determination, but also single particle ICP-MS and coupling to hydrodynamic chromatography for detection of dissolved and particulate silver. The bioaccessible fraction in the intestinal phase accounted for 8–13% of the total silver, mainly in the form of dissolved Ag(I) species, with less than 0.1% as silver-containing particles. Despite the additive behaving differently in pig and chicken digestions, the feed matrix played a relevant role in the fate of the silver.

**Supplementary Information:**

The online version contains supplementary material available at 10.1007/s00216-024-05323-8.

## Introduction

The introduction of novel nano additives in the animal feed chain requires relevant nano-specific considerations that provide insights into exposure assessment and hazard characterization of the nanomaterials. In particular, *in vitro*, *in vivo* and toxicological studies should be taken into account to predict the fate and behaviour of nanoparticles. In this framework, the European Food Safety Authority (EFSA), in its updated guidance on risk assessment of nanomaterials [1], proposed a structured approach for conducting safety evaluations of nanomaterials in food and feed in relation to human and animal health, providing practical recommendations to conduct relevant studies, tests and methods for the identification and characterization of toxicological hazards. The pathway suggested by EFSA is based on investigating nanomaterials following a scheme of five steps. Once the nanomaterial has been characterized, the second step consists in the evaluation of the degradation/dissolution rate of the nanomaterial under representative conditions of the gastrointestinal tract through *in vitro* digestion models. At this point, if the nanomaterial is rapidly or fully dissolved, it follows the standard safety assessment of conventional/non-nanomaterials. Otherwise, further steps of nano-specific testing from the recommended scheme must be followed. The third step involves *in vitro* genotoxicity testing, which implies the assessment of cellular uptake, gene mutation, and DNA aberrations. If the nanomaterial is found non-toxic in this step and shows a high dissolution/degradation rate during the previous one, then the following step of the approach might be argued and waived. The fourth step is an *in vivo* study, which is recommended for the assessment of the nanomaterial absorption, tissue distribution, accumulation and elimination, as well as dose-finding. If the nanomaterial accumulates in organs, further testing is required, leading to the fifth step of the approach. This last step includes more targeted and specific *in vivo* studies, such as human and toxicity studies.

Antibiotics have been used as feed additives to improve the animal husbandry industry for years. They kill virulent bacteria in the digestive tract, leading to an increase in the nutrient absorption and hence, an increase in the animal performance [2]. Nevertheless, this practice may cause the development of antimicrobial resistance. For this reason, the use of antibiotics as growth promoters was banned by the European Union in 2003 [3], leading to an increased interest in the research of potential alternatives. In this sense, silver nanoparticles (AgNPs), as well as dissolved silver (Ag(I)), have proven to have antimicrobial properties [4]. Application of AgNPs includes their incorporation in chicken [5–8] and pig [2, 9, 10] feeds as an alternative to the use of antibiotics to improve animal growth. Fondevila et al. [9] reported that low doses of AgNPs added to pig feed reduced coliform bacteria load in the intestine and improved the daily intake and growth of piglets. Due to their antimicrobial properties, silver doped silica nanoparticles were also added to chicken diet under a dose level up to 4 mg kg^−1^ as a safe growth promoter and a promising alternative to antibiotics [11]. AgNPs showed high antimicrobial activity against the pathogens of poultry farm water and disinfection potential even though 90% of the nanoparticles were aggregated or sedimented in water [7]. Several authors have demonstrated the antimicrobial activity of AgNPs against pathogens present in poultry that may pose a risk to human health: chickens treated with AgNPs showed decreased mortality and lower colonization when infected with *Clostridium perfringens* [6], whereas the addition of 15 nm AgNPs to poultry drinking water reduced the presence of *Escherichia coli*, lowering chicken mortality [7]. On the other hand, the presence of silver traces in chicken meat and organs fed with AgNPs was reported to be harmless to human consumption [12], as well as their addition to drinking water [13].

The study of the fate of a nanomaterial after its ingestion to assess its absorption through the intestine is one of the steps of its risk assessment. Although different *in vitro* gastrointestinal digestion protocols to study the transformations of AgNPs through the gastrointestinal tract are described in the literature, these are only based on human models, which consist of three-step digestibility assays (oral, gastric, and intestinal digestion processes) [14–19]. During the digestion process, nanoparticles can suffer different chemical and physical transformations depending on the medium they are in. These transformations affect the behaviour and toxicity of silver, as well as the bioaccessibility of the element in the intestine, where it is mainly absorbed. In a recent review by Qi et al. [20], the main transformations of orally ingested AgNPs that might occur during human *in vitro* digestion have been discussed in detail. In this work, authors summarized changes that AgNPs could undergo, including agglomeration/aggregation [16, 17] caused by the chloride content of the gastric fluid, oxidative dissolution [18, 19] related to the acidic pH and the presence of oxygen, sulfuration resulting from the interaction with sulfur-containing molecules after the food fermentation, as well as the corona formation around AgNPs due to the gastrointestinal enzymes and food components. The authors also outlined the confusion faced in many articles about what actually happens in the *in vitro* digestion due to the difficulty of recreating the conditions of the gastrointestinal tract and also because it is not clear whether the silver species transformed/produced during the digestion are absorbed by the body or not. However, not only the behaviour of AgNPs has been studied, but also the transformations of Ag(I) in the presence of human simulated digestion fluids [14, 15].Walczak et al. [15] reported the presence of silver-containing particles after the intestinal step using single particle inductively coupled plasma mass spectrometry (SP-ICP-MS), indicating the formation of *de novo* silver particles from Ag(I), as a result of the precipitation of Ag(I) in presence of chloride. Likewise, Abdelkhaliq et al. [14] confirmed the formation of silver particles in the last step of the gastrointestinal simulated digestion, as well as the aggregation and adsorption of 50 nm AgNPs in the digestive matrix. The fate and behaviour of the AgNPs are not only affected by the digestion fluids but also by the food matrix [15, 21–23], since the latter can change the ionic strength or the pH of the digestion fluids. Thus, the EFSA Guidance recommends the study of the impact of food components on the fate of nanomaterials, as changes in the environment of the AgNPs could lead to changes in their behaviour. In the study by Walczak et al. [15], differences in the fate of AgNPs and Ag(I) were found during the gastric and intestinal steps due to the presence of proteins. In the absence of proteins, AgNPs were oxidized and dissolved in the last step, whereas the formation of AgNPs agglomerates during the second step and subsequent deagglomeration in the last one was observed in the presence of proteins. Ag(I) bound to proteins in the intestinal step was also proved in this study.A similar behaviour was observed by Laloux et al. [21], who applied an *in vitro* human digestion to follow the fate of AgNPs in a standard food matrix. Kästner et al. [22] studied the effect of different food matrices (skimmed milk powder, oil and starch), showing that skimmed milk powder stabilized the NPs, whereas oil and starch changed their size distribution, forming aggregates of particles. On the contrary, Lichtenstein et al. [23] revealed that the aggregation of the NPs only happened in the absence of food components, meanwhile in presence of the food, the NPs were protected against the formation of aggregates/agglomerates, most probably because of the non-ionic components, which prevented their formation in acid conditions. Ramos et al. [24] highlighted the different behaviour of 40 nm AgNPs standard in comparison with chicken meat spiked with 50 nm AgNPs. The authors reported that 97% of silver was measured in the intestinal fluid when 40 nm AgNPs were *in vitro* digested, whereas only 57% of silver was recovered from the spiked meat. On the other hand, the detection of silver nanoparticles with smaller sizes was also reported, as well as their dissolution along the digestion steps. However, only SP-ICP-MS was used for the detection of particulate silver and the composition of the particles detected was not confirmed, thus reporting only partial distributions that might correspond to particles containing silver rather than metallic AgNPs.

Whereas harmonized *in vitro* protocols for simulating human digestion have become available as a result of international consensus [25–27], this is not the case for animal digestion. Egger et al. [28] developed an *in vitro* digestion method for pigs, based on the standardized *in vitro* digestion for humans developed by Minekus et al. [27] The *in vitro* digestion method was evaluated in an inter-laboratory study [26] and validated by comparing it with an *in vivo* experiment [28], concluding that the *in vitro* digestion method could be used as a robust and simple tool to study the gastrointestinal digestion processes in pigs. Different *in vitro* methods have been used to simulate the digestion of proteins [29], wheat [30] and triticale [31] in chickens, as well as common poultry feed ingredients (maize, soybean meal, sunflower cake and de-oiled rice) [32]. Yegani et al. [31] developed an *in vitro* digestion method to simulate feed digestion in chickens, where incubation times and enzyme concentrations were optimized. Despite the proposal of using silver-based nanomaterials in feed additives, *in vitro* digestion studies for animals are scarce*.* Abad-Alvaro et al. [10] studied the fate of the AgNPs in weaned piglets but the protocol involved only gastric and intestinal steps, observing the formation of silver chloride particles during the gastric step and their subsequent dissolution in the intestinal step.

In this study, a nanosilver material called silver-kaolin (Ag-kaolin) was risk-assessed under the scope of the EFSA Guidance [1]. A platform of analytical techniques, which includes ICP-MS, SP-ICP-MS and hydrodynamic chromatography coupled to ICP-MS (HDC-ICP-MS) was used for the assessment of the fate and transformations of silver from the Ag-kaolin additive during the *in vitro* gastrointestinal digestion simulated for chickens and pigs. Although different *in vitro* gastrointestinal digestion protocols are described in the literature to study the transformations of AgNP through the gastrointestinal tract, all of them are intended for human digestion. No studies about the fate of a silver based nanomaterial in real animal food during in vitro digestion of pigs and chickens have been conducted yet to the best of our knowledge. For validation purposes, the same *in vitro* digestion procedures were applied to standards of Ag(I) and AgNPs spiked to control feed to study the behaviour of these species along the different steps of both *in vitro* digestion procedures.

## Materials and methods

### Instrumentation

A Perkin-Elmer NexION 2000B ICP mass spectrometer (Toronto, Canada) was used in conventional and single particle detection modes. The sample introduction system consisted of a baffled cyclonic spray chamber and a concentric nebulizer (Meinhard, Colorado, USA). The instrument was tuned daily to obtain maximum ^107^Ag sensitivity, using a 1 μg L^−1^ Ag(I) standard solution. Syngistix™ Nano-Application module version 2.5 (Perkin Elmer, Toronto, Canada) was used for data acquisition and processing in single particle mode. Table S1 in Supporting Information (SI) summarizes the experimental conditions used in both conventional and single particle modes.

For HDC-ICP-MS, a high-performance chromatographic system Waters 2796 Bioseparations module (Waters Corporation, Milford, USA) coupled to a Perkin Elmer Sciex ELAN DRC-e ICP mass spectrometer (Toronto, Canada) was used. HDC separations were performed with a PL-PSDA Type 1 column (Agilent Technologies, Germany) with a nominal separation range of 5–300 nm, a length of 80 cm and an internal diameter of 7.5 mm. The sample flow rate used was 1.6 mL min^−1^. The outflow of the column was delivered directly to the nebulizer of the spectrometer which consisted of a glass concentric slurry nebulizer with a cyclonic spray chamber (Glass Expansion, Melbourne, Australia). The instrument was tuned daily to obtain maximum ^107^Ag sensitivity, using a 10 μg L^−1^ Ag(I) standard solution.. The experimental conditions of the HDC and the ICP-MS are shown in Table S1.

A PerkinElmer 2380 flame atomic absorption spectrophotometer (FAAS) (PerkinElmer, Norwalk, Connecticut, USA) was used for the determination of of total silver content released from the Ag-kaolin material in ultrapure water. A wavelength of 328.1 nm and an acetylene/air flame were used.

### Reagents and materials

Aqueous Ag(I) solutions were prepared from a standard stock solution of 994 ± 3 mg L^−1^ (Panreac, Barcelona, Spain) by dilution in ultrapure water with a resistivity of 18.2 MΩ cm (Milli-Q Advantage, Molsheim, France) by accurately weighing (± 0.1 mg). Diluted suspensions of silver nanoparticles were prepared from commercial suspensions of citrate-stabilized (0.02 mg mL^−1^) silver nanoparticles of 10.3 ± 2.1, 19.1 ± 3.6, 39 ± 5 and 59 ± 6 nm (Nanocomposix, San Diego, USA). Aqueous gold solutions were prepared from a standard stock solution of 999 ± 4 mg L^−1^ (Panreac, Barcelona, Spain) by dilution in ultrapure water. Ultra-uniform gold nanoparticles (PEG-carboxil 0.8 kDa surface) of 47.8 ± 1.8 nm (Nanocomposix, San Diego, USA) with a concentration of 52 mg L^−1^ were used for transport efficiency calculation. Nitric acid (69/70%, J.T. Baker, Phillipsburg, USA) and hydrochloric acid (69/70%, J.T. Baker, Phillipsburg, USA) were used for acid digestions of feeds. 3kDa Nanosep Pall centrifugal ultrafilters (Nanosep, Pall, Ann Arbor, USA) were used for ultrafiltration to isolate the ionic fraction of silver. First, ultrafilters were washed by centrifugation with 500 μL of ultrapure water. Then, the devices were washed again to avoid any potential contamination. Afterward, 500 μL of the supernatants of Ag-kaolin suspension were centrifuged at 15000g for 30 min at 20 ºC (Heraeus Multifuge X1R, Thermo Fisher Scientific, Walthman, USA.). The ultrafiltration recovery was 93.3 ± 1.6%.

The mobile phase used for the HDC separation contained 0.45 mM sodium dodecyl sulphate (SDS) (Bio-Rad Laboratories, California, USA) and 1 mM D-Penicillamine (PA) (Sigma Aldrich, St. Louis, USA) in ultrapure water. Nitric acid (69/70%, J.T. Baker, Phillipsburg, USA) solution was used to reach pH 7.5.

The nanosilver-based material (Ag-kaolin) contained AgNPs adsorbed on kaolinite microparticles, and it was developed to be used as a growth promoter and antibiotic substitute in animal feed. The antibacterial activity and the *in vitro* genotoxicity of the Ag-kaolin were conducted in previous studies [33, 34]. The material was found to have an anti-bactericidal activity against a wide variety of bacterial strains and did not cause DNA damage or gene mutation in mouse lymphoma cells. The nanomaterial was provided by Laboratorios Enosan S.L. (Spain). Ag-kaolin was added to the feeds used in this study at 0.2% (*w*/*w*). The composition of the pig and chicken feeds is shown in Tables S2 and S3, respectively.

For the pig *in vitro* digestion, pepsin from porcine gastric mucosa stock of 3200–4500 U mL^−1^ protein (Sigma Aldrich, St. Louis, USA); pancreatin from porcine pancreas based on trypsin activity (Sigma Aldrich, St. Louis, USA) and bile bovine B3883 (Sigma Aldrich, St. Louis, USA) were used to prepare the correspondent enzyme solutions. Potassium chloride (KCl) (Sigma Aldrich, St. Louis, USA), potassium dihydrogen phosphate (KH_2_PO_4_) (Sigma Aldrich, St. Louis, USA), sodium bicarbonate (NaHCO_3_) (Sigma Aldrich, St. Louis, USA), sodium chloride (NaCl) (VWR Chemicals Prolabo, New Jersey, USA), magnesium chloride (MgCl_2_·6H_2_O) (Fisher, Leicestershire, UK), ammonium carbonate ((NH_4_)_2_CO_3_) (Fluka BioChemika, Buchs, Switzerland), calcium chloride (CaCl_2_·2H_2_O) (Fisher, Leicestershire, UK) and Milli-Q water (Milli-Q Advantage, Molsheim, France) were used to prepare the simulated fluids. Table S4 shows the concentrations of the different salts and enzymes to prepare the digestion fluids. Sodium hydroxide (NaOH) (Prolabo, Barcelona, Spain) and hydrochloric acid (37%, J.T. Baker, Phillipsburg, USA) were used to adjust the pH.

For the chicken *in vitro* digestion a pepsin solution (25 mg mL^−1^; P-7000, Sigma-Aldrich) and a porcine pancreatin solution (100 mg mL^−1^, P-1750, Sigma Aldrich) were used as enzymes. Moreover, sodium phosphate monobasic monohydrate (NaH_2_PO_4_) (Sigma-Aldrich, Germany), sodium phosphate dibasic (Na_2_HPO_4_) (Sigma-Aldrich, Germany) and Milli-Q water (Milli-Q Advantage, Molsheim, France) were used to prepare the simulated fuids. Table S5 shows the composition of gastro-intestinal digestion fluids of the chicken in vitro digestion. Sodium hydroxide (NaOH) (Prolabo, Barcelona, Spain) and hydrochloric acid (37%, J.T. Baker, Phillipsburg, USA) were used for pH adjustment.

### Procedures

#### Determination of total silver in feeds

For the determination of the total silver content in feeds, the procedure by Jiménez-Lamana et al.[35] was used. One hundred mg of feed were weighed and put into digestion vessels and 7 mL of HNO_3_ and 3 mL of HCl were added. The digestion was performed in a microwave oven (Mars 6 CEM, Charlotte, North Carolina, USA) at 200 °C and 800 psi for 30 min. After cooling, the content of the vessels was transferred to polyethylene tubes and made up to 50 mL with 3% HCl (*v/v*). ICP-MS was used for the determination of total silver. The calibration curve was performed from 1 to 15 µg L^−1^ of Ag(I) and 10 µg L^−1^ of Rh was used as the internal standard.

#### *In vitro* digestion of pig and chicken feeds

##### Pig feed

The *in vitro* digestion procedure followed was described by Minekus et al. [27] first, and later optimized for pigs by Egger et al. [26] and consisted of three steps: 1) oral, 2) gastric, and 3) intestinal. An amount of 0.5 g of feed was accurately weighed and put into polyethylene tubes. For the oral step, 3.5 mL of simulated salivary fluid (SSF) and 0.025 mL of 0.3 M CaCl_2_·2H_2_O solution were added and then filled with ultrapure water up to 10 mL. The tubes were vortexed and sonicated (200 W, 1 min, room temperature) to ensure the homogenization of the mixture. Then, the suspension was incubated with shaking (99 rpm) for 2 min at 37 °C in darkness. For the gastric step, 10 mL of oral suspension was mixed with 7.5 mL of simulated gastric fluid (SGF), 0.005 mL of 0.3 M CaCl_2_·2H_2_O solution and 1.6 mL of porcine pepsin solution. 1 M HCl solution was used to get pH 3.0 and ultrapure water was added to reach a final volume of 20 mL. The suspensions were vortex, sonicated and then, incubated with shaking (99 rpm) for 2 h at 37 °C in darkness. For the intestinal step, 20 mL of gastric solution was blended with 11 mL of simulated intestinal fluid (SIF), 0.04 mL of CaCl_2_·2H_2_O solution, 2.5 mL of fresh bile and 5 mL of pancreatin solution. 1 M NaOH solution was used to get pH 7 and ultrapure water was added to reach a final volume of 40 mL. The suspension was incubated with shaking (99 rpm) for 2 h at 37 °C in darkness. At the end of each step, suspensions were centrifuged (4700 g, 17 min, 20 °C) to remove Ag-kaolin particles larger than 1 µm. Supernatants were stored at 4 ºC in the dark for further analyses. All experiments were performed in triplicate.

Moreover, this *in vitro* digestion process was applied to 5 mg L^−1^ of Ag(I) and/or 1 mg L^−1^ of 20 nm and/or 40 nm AgNPs to study the behaviour of these silver species along the different digestion steps. Likewise, pig control feed was also spiked with 5 mg L^−1^ of Ag(I) and 1 mg L^−1^ of 20 nm AgNPs and subjected to this *in vitro* digestion process to figure out the effect of the feed matrix on the behaviour of Ag(I) and AgNPs. Supernatants related to the digestion of Ag(I) (with and without feed matrix) were further analyzed by ICP-MS, SP-ICP-MS and HDC-ICP-MS. On the other hand, supernatants from the digestion of 40 nm (with and without feed matrix) were studied by ICP-MS and SP-ICP-MS. Finally, supernatants from the digestion of 20 nm AgNPs (with and without matrix) were only analyzed by HDC-ICP-MS.

##### Chicken feed

An *in vitro* two-step digestion procedure (gastric and intestinal) based on previous protocols for *in vitro* digestibility of feedstuffs was used [31, 36]. Table S5 summarizes the composition of the simulated digestion fluids. Half a gram of feed was accurately weighed and put into polyethylene centrifugation tubes. For the gastric step, 25 mL of phosphate buffer (0.1 M, pH 6.0) was added to each sample. Tubes were gently mixed and 10 mL of HCl (0.2 M) were added to get pH 2.0. Subsequently, 1 mL of a freshly prepared porcine pepsin solution was added. Tubes were vortexed for 1 min to ensure homogenization of the mixture, then bath sonicated for 1 min (200 W, 1 min, room temperature) to avoid NPs aggregation. Mixtures were placed in an incubator at 41 °C and shaken at 100 rpm for 2 h in darkness. After the gastric step, 10 mL of phosphate buffer (0.2 M, pH 6.8) were added, as well as 0.6 M NaOH to get pH 6.8. Tubes were gently shaken and 1 mL of a freshly prepared porcine pancreatin solution (100 mg mL^−1^) including amylase, lipase and protease was added. Tubes were vortexed (1 min) and sonicated (1 min), then incubated at 41 °C with shaking at 100 rpm for 4 h in darkness. At the end of each step, suspensions were centrifuged (4700 g, 17 min, 20 °C) to remove Ag-kaolin particles larger than 1 µm. Supernatants were stored at 4 ºC in the dark for further analyses. All experiments were performed in triplicate. Moreover, this *in vitro* digestion process was applied to 5 mg L^−1^ of Ag(I) and/or 1 mg L^−1^ of 40 nm AgNPs to study the behaviour of these silver species along the different digestion steps. Likewise, chicken control feed was also spiked with 5 mg L^−1^ of Ag(I) and/or 1 mg L^−1^ of 40 nm AgNPs and subjected to this *in vitro* digestion process to figure out the effect of the feed matrix on the behaviour of Ag(I) and AgNPs. Supernatants were further analysed by ICP-MS, SP-ICP-MS and HDC-ICP-MS.

#### Analysis of digestion fluids

The total silver content in the supernatants from the different digestion steps was determined by ICP-MS, using Rh as an internal standard. Calibrations were performed using Ag(I) standards prepared in 1% (*v/v*) HNO_3_, since no significant differences in sensitivity were observed when calibrating in the digestion fluid matrices (data not shown).

SP-ICP-MS and HDC-ICP-MS were used to obtain information about dissolved and particulate silver. For SP-ICP-MS analysis, the supernatants were diluted with ultrapure water, according to their silver concentrations [37], and measured using a dwell time of 100 µs and an acquisition time of 50 s for the standards and spiked samples, which had to be increased up to 300 s for the feed samples to improve the detectability for particles. Dissolved silver was determined from the baseline intensity of the time scans and calibration with Ag(I) standards. Silver mass per particle and the corresponding distributions were determined from the intensity of the individual particle events by considering the analyte transport efficiency, the sample flow rate and the Ag(I) calibration [38]. The transport efficiency was calculated by the size method [38] using Au nanoparticle standard. The sample flow rate was determined gravimetrically.

## Results and discussion

### Silver-kaolin additive characterization

The Ag-kaolin additive consisted of spheroidal metallic silver nanoparticles adsorbed on kaolin microparticles, as shown in Fig. S1 of SI. Information about the material can be found elsewhere [34] and Table S6 summarizes additional physicochemical parameters. The silver nanoparticles showed diameters in the range of 10 to 90 nm, with a mean diameter of 27 nm. The total silver content was 8.32 ± 0.35 mg g^−1^. Dispersion of the additive in water showed the rapid sedimentation of particles over ca. 2 µm, remaining polydisperse particles with a mean hydrodynamic diameter of 1 µm in suspension within 30 min, when measuring by dynamic light scattering. For this reason, a centrifugation step to remove kaolin microparticles larger than ca. 1 µm was included to isolate the colloidal fraction of the additive in the supernatants of the suspensions.

Despite the insolubility of kaolin in water, the behaviour and solubility of the metallic silver nanoparticles adsorbed in this Ag-kaolin additive was considered. After 1 h, 32% of the silver was released from a 1 g L^−1^ dispersion of additive, remaining the silver concentration (2.69 ± 0.18 mg L^−1^) nearly constant for longer times, as it can be seen in Fig. S2. When dispersed in water, the additive could release silver in its particulate and ionic forms, the latter coming from the oxidation of the nanoparticles themselves or most likely from the dissolution of a complex silver oxide outer coating present in metallic silver nanoparticles [41]. The different silver species in Ag-kaolin leachates in water were determined by SP-ICP-MS and HDC-ICP-MS. The limits of detection of SP-ICP-MS for dissolved silver, silver mass per particle and particle number concentration were calculated as reported in Laborda *et al.* [39], as 0.7 ng L^−1^, 55 ag per particle and 1.4 × 10^6^ particles L^−1^, respectively. Quantification of the silver species by HDC-ICP-MS was carried out using Ag(I) standards stabilized with 1 mM PA [40]. Size (hydrodynamic diameter) calibration was performed using silver nanoparticles standards with nominal sizes between 10 and 60 nm and plotting their retention time vs. nominal diameter. The column recovery for Ag(I) was 99.9 ± 1.7%. Detection and quantification limits were calculated from the standard deviation of the baseline and the peak height of the Ag(I) peak as reported in Jimenez et al. [40] as 0.75 μg L^−1^ and 2.5 μg L^−1^, respectively.

The analysis of the supernatants by HDC-ICP-MS did not allow the detection of particulate forms of silver, just obtaining a single peak corresponding to dissolved silver (Fig. S3 in SI). However, silver-containing particles were detected by SP-ICP-MS at silver concentrations of ca. 1 ng L^−1^. The silver content per particle was in agreement with the size of the nanoparticles originally present in the additive (Fig. S4 in SI), although the detection of kaolin particles containing silver could not be discarded. In any case, the analysis of the supernatants by HDC-ICP-MS and SP-ICP-MS, as well as after their ultrafiltration followed by FAAS analysis (Table S7), revealed that silver was mainly released as dissolved Ag(I) forms. Using SP-ICP-MS, less than 4 × 10^–5^% (*w/w*) of silver was detected as particles and the rest of the released silver was found in the form of Ag(I). Similar results were obtained by ultrafiltration and FAAS where 99 ± 8% (*w/w*) of the total silver was released as dissolved Ag(I) as it can be seen in Table S7 of SI. This percentage of particulate silver and Ag(I) was calculated with respect to the total silver released from Ag-kaolin in water.

### Behaviour of silver species along the pig and chicken in vitro digestions

Although the EFSA Guidance only emphasizes the importance of the intestinal step in assessing the degradation of nanomaterials [1], it is essential to previously simulate both the oral and gastric steps (only the gastric for chickens) to provide valuable information on the transformations and fate of silver. The *in vitro* digestion procedures for pigs and chickens described in Sect. 2.3.2 were applied to Ag(I) and AgNPs standards with and without feed matrix. Silver present in the supernatants of the intestinal step was considered as the bioaccessible fraction.

As a first approach, the behaviour of Ag(I) and 40 nm AgNPs during the *in vitro* digestions was studied through the determination of the total silver content in the supernatants of each digestion step by ICP-MS. Table 1 summarizes the silver content, quoted as recovery, found in the supernatants from the silver standards directly subjected to *in vitro* digestion (no feed matrix), or previously spiked in control feeds.

**Table 1:** Recovery of Ag(I) and 40 nm AgNPs in each *in vitro* gastrointestinal digestion step for pigs and chickens with and without feed matrix determined by ICP-MS (mean ± standard deviation, n = 3)

**Animal**	**Total silver recovery (%)**					
	**Oral**		**Gastric**		**Intestinal**	
	**Ag(I)**	**AgNPs**	**Ag(I)**	**AgNPs**	**Ag(I)**	**AgNPs**
**Without feed matrix**						
Pig	105.3 ± 7.9	87.1 ± 5.7	31.2 ± 1.8	68.0 ± 18.1	80.8 ± 1.6	71.5 ± 3.0
Chicken	-	-	65.4 ± 1.0	8.7 ± 0.1	72.9 ± 9.4	30.5 ± 0.1
**With feed matrix**						
Pig	13.2 ± 1.2	12.3 ± 1.1	7.3 ± 0.2	7.9 ± 1.0	8.0 ± 0.1	83.2 ± 5.1
Chicken	-	-	11.1 ± 0.1	39.0 ± 6.4	57.0 ± 1.0	55.1 ± 3.1

The recovery of Ag(I) in the gastric step was in the range of 30 and 65% for pig and chicken digestion processes, respectively; while in the intestinal step, these values increased to 70–80%. The low recoveries found for Ag(I) in the gastric step are associated with the presence of chloride and the precipitation of silver as AgCl, as reported in bibliography [10, 19], whereas its redissolution during the intestinal step would be favoured by the presence of enzymes and the pH rise, resulting in the increase of the bioaccessible fraction both for pigs and chickens. On the other hand, differences in total silver recoveries were observed in the *in vitro* digestion of 40 nm AgNPs standards depending on the animal protocol. In the case of pigs, recoveries around 70% were obtained for both the gastric and intestinal steps. However, recoveries below 10% in the gastric step, which increased up to 30% in the intestinal one were observed for chicken.

**Fig. 1:**
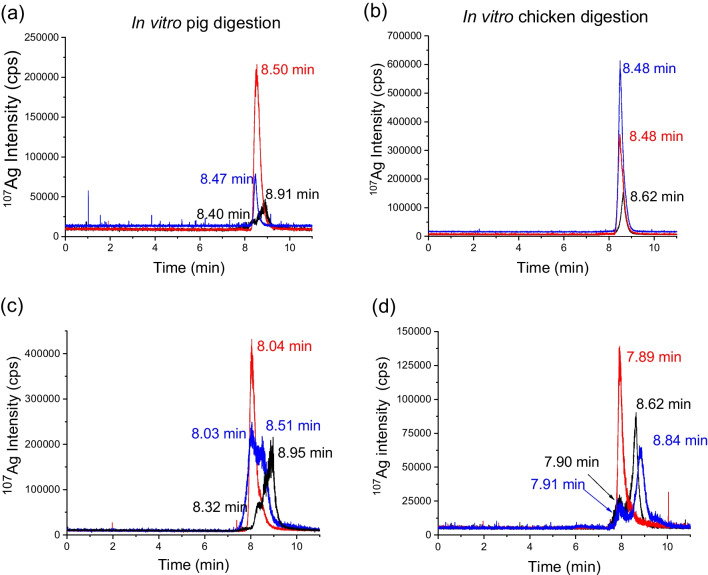
HDC-ICP-MS chromatograms of Ag(I) (**a** and **b**) and AgNPs (**c** and **d**) standards (red), subjected to *in vitro* digestion (blue) and spiked in control feed and subjected to *in vitro* digestion (black). Analysis of supernatants from the intestinal step *of in vitro* pig (**a** and **c**) and chicken (**b** and **d**) digestions. AgNPs: 20 nm in pig digestion (**c**) and 40 nm in chicken digestion (**d**)

To follow the transformation of the silver species at the different steps of the gastrointestinal digestions, the supernatants were also analyzed by HDC-ICP-MS and SP-ICP-MS. SP-ICP-MS was used to check the presence of particulate silver, whereas HDC-ICP-MS could differentiate between dissolved and nanoparticulate silver due to their different elution time. Detectable amounts of particulate forms of silver derived from the *in vitro* digestion of Ag(I) were not found in any of the supernatants of the intestinal digestion fluids analyzed by SP-ICP-MS (data not shown), or by HDC-ICP-MS (Fig. 1 a, b). On the other hand, in the case of the *in vitro* digestion of 40 nm AgNPs, the presence of dissolved silver was detected by HDC-ICP-MS in all digestion steps, confirming the oxidative dissolution of AgNPs (Fig. 1 c, d for the intestinal step and Fig. S5 in SI for the rest of the steps). Although AgNPs were not detected by HDC-ICP-MS in the pig gastric fluid (Fig. S5- b), they were observed in the intestinal step (Fig. 1c), in agreement with chloride-mediated agglomeration of the nanoparticles in the gastric step followed by deagglomeration in the intestinal one, as reported by different authors for human *in vitro* digestion [15, 18, 23].

In the presence of feed matrix, total silver recoveries for Ag(I) in the different steps of the pig *in vitro* digestion were in the range of 8–13%, much lower than the previous results without matrix. In the case of the chicken *in vitro* digestion, recoveries were also lower although higher than for pigs, with a bioaccessible fraction close to 60%. These results suggest that the feed matrix contributes to retain Ag(I) in the solid fraction decreasing its bioaccessibility. In the case of AgNPs, the presence of the feed matrix contributed to an increase in the bioaccessible fraction up to 83 and 55% for pig and chicken, respectively. However, a relevant part of the nanoparticles was oxidized to Ag(I), as observed by HDC-ICP-MS (Fig. 1 in black). Moreover, the presence of the feed matrix affected the retention times of the different species. The relevance of the food/feed matrix has also been pointed out by different authors in the case of human *in vitro* digestion [21–24].

The different behaviour of silver species along the *in vitro* digestion for pigs and chickens could be explained by the different conditions in each procedure, namely incubation time (6 h for chickens and 4 h for pigs), temperature (41 °C for chickens and 37 °C for pigs), the use of different pepsins and the absence of bile in the chicken intestinal fluid.

### Determination of total silver in feeds

The total content of silver in the different feeds under study is shown in Table S8 of SI. Three replicates of each type of feed were analyzed by ICP-MS, following the acid digestion procedure described in Sect. 2.3.1. Control feed showed silver content of 0.1 mg kg^−1^ or lower, whereas the content of silver in both feeds supplemented with Ag-kaolin was ca. 12 mg kg^−1^. The detection (LOD) and the quantification limits (LOQ) were calculated as 3 and 10 times the standard deviation of 10 procedural blanks divided by the slope of the calibration curve, with values of 16 and 54 µg L^−1^, respectively. Quantitative recoveries in the range of 98–100% were obtained from the analysis of spiked samples and control feeds spiked with Ag(I). Procedure recoveries ranged from 88.2 to 92.6%, whereas recoveries from spiked pig and chicken feed were 83.8 ± 11.3% and 104 ± 0.30%, respectively.

### Fate of silver during the in vitro digestion of feeds supplemented with Ag-kaolin

Once the behaviour of different silver species along the different steps of pig and chicken *in vitro* digestions was studied, these procedures were applied to feeds supplemented with Ag-kaolin. Silver could be present in the supernatants as metallic silver nanoparticles adsorbed on kaolin microparticles (below ca. 1 µm), as free metallic silver nanoparticles, as dissolved forms of Ag(I) from the oxidation of the silver nanoparticles, as well as other species from the transformation of the previous ones (e.g., silver chloride nanoparticles, aggregates of metallic silver nanoparticles, other Ag-containing nanoparticles).

Total silver content of the different digestion steps was determined by ICP-MS. Moreover, as in the case of previous studies with silver standards, the supernatants were also analyzed by HDC-ICP-MS and SP-ICP-MS to get a better insight into the nature of the different silver species released from the feeds supplemented with Ag-kaolin. Table 2 summarizes the fraction of silver released from the supplemented feeds found by ICP-MS, SP-ICP-MS and HDC-ICP-MS. The bioaccessible silver fraction at the end of the intestinal step accounted for less than 10%, both for pig and chicken simulated digestions. It should be noted that the intestinal fluids contributed to increase the bioaccessible fraction with respect to the silver released at the end of the gastric step, which was below 3%. Total silver by SP-ICP-MS and HDC-ICP-MS during the intestinal step was in the range of 8 to 13% for pigs and 12% for chickens in agreement for all techniques.

**Table 2:** Released silver fraction rom feeds supplemented with Ag-kaolin in each *in vitro* gastrointestinal digestion step for pigs and chickens determined by ICP-MS, SP-ICP-MS and HDC-ICP-MS (mean ± standard deviation, n = 3)

Animal digestion	Total silver released (%)								
	**Oral**			**Gastric**			**Intestinal**		
	ICP-MS	SP-ICP-MS	HDC-ICP-MS	ICP-MS	SP-ICP-MS	HDC-ICP-MS	ICP-MS	SP-ICP-MS	HDC-ICP-MS
**Pig**	1.84 ± 0.07	2.13 ± 0.25	2.20 ± 0.17	1.78 ± 0.53	2.67 ± 0.64	3.26 ± 0.34	8.33 ± 0.01	13.5 ± 1.20	8.95 ± 0.48
**Chicken**	-	-	-	2.92 ± 0.70	2.23 ± 0.90	-	9.05 ± 2.00	12.1 ± 3.1	12.1 ± 3.9

### Occurrence of silver species during in vitro digestion of chicken feed

**Fig. 2:**
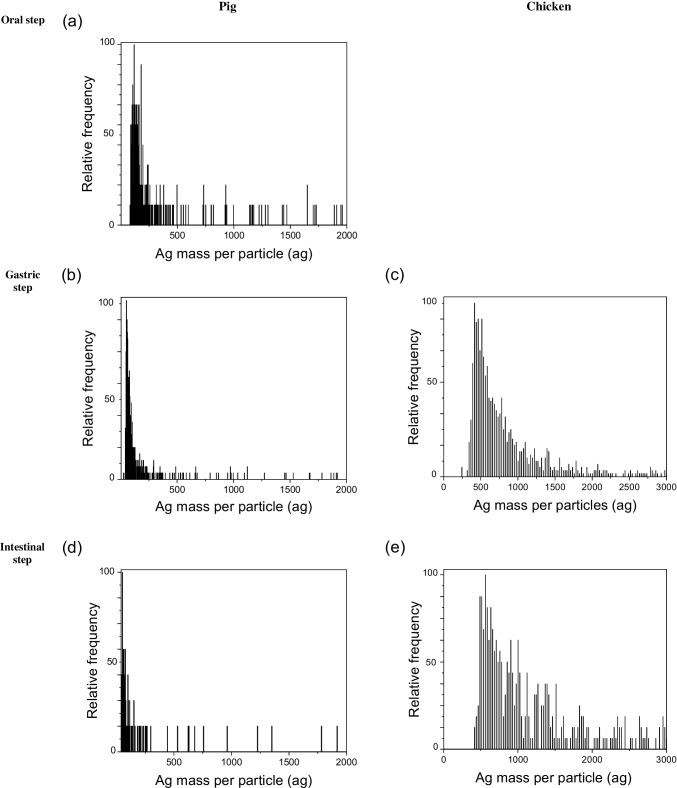
SP-ICP-MS mass per particle distribution of silver-containing particles released during the *in vitro* digestion of pig and chicken feed supplemented with Ag-kaolin. (**a**) oral step, (**b**, **c**) gastric steps, (**d**, **e**) intestinal steps

Figure 2 (c and e) shows the mass per particle distribution of silver-containing particles detected by SP-ICP-MS for the analysis of the supernatants from the gastric and intestinal steps in chicken. Silver particles concentrations corresponded to 2 and 7 ng L^−1^, respectively, which accounted for 0.02 and 0.06% (*w/w*) of the total silver released in the respective steps. In addition, the dissolved silver determined by this technique (0.3 and 1.5 µg L^−1^) accounted for 2.2 and 12.1% of the total silver released in each step, in fair agreement with the results obtained by ICP-MS (Table 2). These results reveal that bioaccessible silver was released from supplemented feed mainly as dissolved species from the oxidation of the Ag-kaolin additive metallic nanoparticles, although a very small fraction of silver-containing particles was also present. Although the exact nature of the particles cannot be determined by this technique, their mass would be equivalent to spherical silver nanoparticles of 31–34 nm in diameter.

**Fig. 3:**
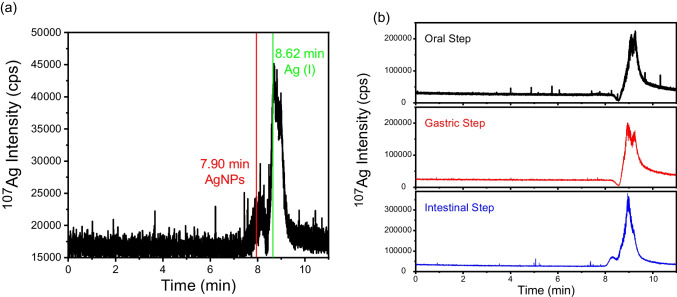
HDC-ICP-MS chromatograms obtained for the in vitro digestion supernatants of (**a**) chicken feed (intestinal step) and (**b**) pig feed (oral, gastric and intestinal steps) supplemented with Ag-kaolin. Retention times of 40 nm AgNPs (in red) and Ag(I) (in green) correspond to their elution in spiked control feed

Due to the low amount of silver released from the gastric step, only the intestinal fluid could be analyzed by HDC-ICP-MS. Figure 3a shows the chromatogram obtained from the supernatant of the intestinal step, which shows a peak at 8.62 min. This retention time is in agreement with the elution time previously observed after the digestion of Ag(I) spiked in control feed matrix (Fig. 1b). Another small peak appeared at ca.7.90 min, which was suspected to correspond to a particulate form of silver; however, its specific nature was unknown. It could correspond to 20 nm AgNPs (t_r_ = 7.92 ± 0.08 min) resulting from the degradation of the original NPs present in the Ag-kaolin nanomaterial or to another particulate silver form. In any case, the HDC-ICP-MS chromatogram confirmed the presence of dissolved silver in the bioaccessible fraction, whose quantification corresponded to 12 ± 4% of the total silver released in the intestinal step, in agreement with the results obtained by ICP-MS and SP-ICP-MS (Table 2). The oxidation of AgNPs to Ag(I) during the gastrointestinal digestion had previously been observed in the analysis of 40 nm AgNPs and control feeds spiked with 40 nm AgNPs (Fig. 1d).

### Occurrence of silver species during in vitro digestion of pig feed

Figure 2 (a, b and d) shows the mass per particle distribution of silver-containing particles detected by SP-ICP-MS for the analysis of the supernatants from the oral, gastric and intestinal steps in pigs. Because of the low levels of particulate forms of silver in the different digestion steps, acquisition times had to be increased from 1 to 5 min. Particles silver concentrations corresponded to 1 and 2 pg L^−1^ in the oral and gastric steps, and were found to be below the limit of quantification in the intestinal one, which accounted for around 0.00001% (*w/w*) of the total silver released. The most frequent silver mass per particle was ca. 60 ag. In addition, the dissolved silver determined by this technique (0.26, 0.30 and 1.70 µg L^−1^) represented 2.1, 2.7 and 13.5% of the total silver released in each step. These results reveal that, as in the case of chicken, the bioaccessible silver was released from the supplemented feed mainly as dissolved species from the oxidation of the Ag-kaolin metallic nanoparticles. However, the fraction of silver-containing particles was lower in the case of pigs.

The HDC-ICP-MS chromatograms obtained from the supernatants of the different steps of the *in vitro* digestion are shown in Fig. 3b. Peaks from 8.40 up to 9.25 min were obtained. Comparison with chromatograms obtained for the *in vitro* digestion of Ag(I) and 20 nm AgNPs standards with and without feed matrix (Fig. 1a, 1c) confirmed the absence of particulate silver of particulate silver, in agreement with the low levels observed by SP-ICP-MS. Therefore, all the peaks recorded corresponded to Ag(I) species, although delays or splits with respect to the elution of the standards were observed. This could be due to the complexation of the ionic silver with different compounds present in the feed or digestion fluids, along with the different interactions of these species with the stationary phase of the column. Even the two peaks observed from the intestinal step were associated with Ag(I), as it was confirmed by the analysis of spiked control feed (Fig. 1a). The quantification of the chromatograms showed that 2.2, 3.3 and 9.0% of the total silver was released in each step, in fair agreement with the results obtained by ICP-MS (Table 2).

## Conclusions

According to EFSA [1], the assessment of the dissolution/degradation of nanomaterials in representative conditions of the gastrointestinal digestion is one of the steps for the risk assessment of nanomaterials to be used as feed additives. The fate and transformations of silver nanoparticles from Ag-kaolin additive, incorporated as a growth promoter in the feed formulations of chickens and pigs, were studied following two *in vitro* digestion models that mimic the gastrointestinal digestion of these animals. Since the additive consisted of metallic silver nanoparticles adsorbed onto kaolin microparticles, the exposure to the nanoparticles should involve their release from the matrix with minor degradation. Due to the different processes that silver, both metallic and ionic, can undergo under gastrointestinal digestion, the silver present in the intestinal step was considered as the bioaccessible fraction and determined by a number of ICP-MS based methods, which included ICP-MS for total silver determination, but also HDC-ICP-MS and SP-ICP-MS to get insight into the different forms, dissolved and particulate, of silver.

The *in vitro* digestion of feeds supplemented with the Ag-kaolin additive (0.2%) resulted in 8–13% of bioaccessible silver, mainly in the form of dissolved Ag(I) species with less than 0.1% as silver-containing particles, while the rest of silver was present in the solid fraction, as adsorbed or precipitated Ag(I) species, agglomerated/aggregated AgNPs or Ag-kaolin particles. Since the EFSA Guidance considers a cut-off of 12% (mass-based) or less of a nanomaterial present as particles after 30 min of intestinal digestion, a nanospecific risk assessment would not be required for the additive under study. The low bioaccessibility of the silver from the additive is reflected in its low retention in animal tissues, as it has been confirmed by i*n vivo* experiments [42]. Under such conditions, most of the ingested silver is excreted through the faeces [43], and the use of slurries and manure as amendments in agriculture should consider the potential mobilization of silver species in the environment.

### Supplementary Information

Below is the link to the electronic supplementary material.

Supplementary file1 (DOCX 2.48 MB)
