# Supplementary material for: Nanosilver-based materials as feed additives: Evaluation of their transformations along in vitro gastrointestinal digestion in pigs and chickens by using an ICP-MS based analytical platform
Source: Anal Bioanal Chem. 2024 May 22;416(16):3821–33. doi: 10.1007/s00216-024-05323-8 (PMC11180633; doi:10.1007/s00216-024-05323-8)
Supplement: Supplementary file 1 — Supplementary file1 (DOCX 2.48 MB) [file 216_2024_5323_MOESM1_ESM.docx]

**Supplementary material**

**Nanosilver-based materials as feed additives: Evaluation of their transformations along *in vitro* gastrointestinal digestion in pigs and chickens by using an ICP-MS based analytical platform**

Khaoula Ben‑Jeddou, Mariam Bakir, María S. Jiménez*, María T. Gómez, Isabel Abad-Álvaro, Francisco Laborda

Group of Analytical Spectroscopy and Sensors (GEAS), Institute of Environmental Sciences (IUCA), University of Zaragoza, Pedro Cerbuna 12, 50009 Zaragoza, Spain

*Corresponding author: jimenezm@unizar.es

**Table S1**. Instrumental operating conditions.

**Table S2.** Composition of pig feed.

**Table S3.** Composition of chicken feed.

**Table S4**. Composition of the gastrointestinal fluids of the pig *in vitro* digestion.

**Table S5.** Composition of the gastrointestinal juices of the chickens *in vitro* digestion.

**Fig. S1.** FESEM images of the Ag-kaolin additive.

**Table S6.** Properties of the Ag-kaolin additive.

**Fig. S2.** Release of total silver from Ag-kaolin in ultrapure water in function of time.

**Fig. S3.** HDC-ICP-MS chromatograms. Supernatant suspension of Ag-kaolin material (1:2 dilution). concentration: 1 g L^-1^.

**Fig. S4.** Size distribution of the supernatant suspension of Ag-kaolin material (1:50 dilution). Concentration: 1 g L^-1^.

**Table S7**.Release of total and dissolved silver in ultrapure water of a 1 g L^-1^ of a dispersion of Ag-kaolin with the different used techniques (mean ± standard deviation, n=3).

**Fig. S5**. Chromatogram of 20 nm AgNPs and a spike of 20 nm AgNPs on control feed for the oral and gastric steps of pigs *in vitro* digestion of: (a) SSF (b) SGF.

**Table S8**. Total silver concentration in pig and chicken control and supplemented feeds (mean ± standard deviation, n=10).

**Table S1**. Instrumental operating conditions.

| **ICP-MS** |  |
| --- | --- |
| RF power (W) | 1200 |
| Argon gas flow |  |
| Plasma (L min^-1^) | 15 |
| Auxiliary (L min^-1^) | 1.2 |
| Nebulizer (L min^-1^) | 1.02 |
| Sample flow rate (mL min^-1^) | 0.35 |
| Data acquisition parameters |  |
| Dwell time (ms) | 50 |
| Readings per replicate | 1 |
| Isotopes monitored | ^107^Ag, ^109^Ag |
|  |  |
| **SP-ICP-MS** |  |
| RF power (W) | 1200 |
| Argon gas flow |  |
| Plasma (L min^-1^) | 15 |
| Auxiliary (L min^-1^) | 1.2 |
| Nebulizer (L min^-1^) | 1.02 |
| Sample flow rate (mL min^-1^) | 0.35 |
| Data acquisition parameters |  |
| Dwell time (µs) | 100 |
| Readings per replicate | 500,000/3,000,000 |
| Total acquisition time (s) | 50/300 |
| Isotopes monitored | ^107^Ag |
|  |  |
| **HDC-ICP-MS** |  |
| Forward power (w) | 1100 |
| Argon gas flow |  |
| Plasma (L min^-1^) | 15 |
| Auxiliar (L min^-1^) | 1.2 |
| Nebulizer (L min^-1^) | 1.0 |
| Data acquisition parameters |  |
| Sweeps per reading | 1 |
| Readings per replicate |  |
| With column | 6250 |
| Without column | 600 |
| Dwell time (ms) | 50 |
| Acquisition mode | Peak hopping |
| Total acquisition time (min) | 11 |
| Isotopes monitored | ^107^Ag, ^109^Ag |
| **HDC** |  |
| Column | PL-PSA type 1 |
| Flow rate (mL min^-1^) | 1.6 |
| Injection volume (µL) | 50 |
| Mobile phase | 0.45 mM SDS and 1 mM PA |

**Table S2.** Composition of pig feed.

|  | Content  (g kg^-1^) |
| --- | --- |
| Ingredient composition  Maize grain  Barley grain  Soybean meal  Whey powder  Fishmeal LT  Sunflower oil  Dicalcium phosphate  Calcium carbonate  Sodium chloride  Mineral-vitamin mix^1^  Ag-kaolin^2^ | 400  250  200  60  40  15  18  10  3.0  4.0  2.0 |
| Analysed composition  Organic matter  Crude protein  Ether extract  Neutral detergent fibre | 923  189  43  175 |

^1^To give per kg feed: 13,000 IU vit. A; 2500 IU vit. D3; 13 mg vit. E; 1.5 mg vit. K; 5 mg riboflavin; 1 mg tiamin; 2.2 mg vit. B6; 0.02 mg vit. B12; 25 mg niacin; 10 mg calcium pantothenate; 200 mg choline chloride; 110 mg Zn; 50mg Mn; 100 mg Fe; 165 mg Cu;0.5 mg Co; 0.22 mg Se; 0.5 mg I.

^2^ Not included in control feed

**Table S3.** Composition of chicken feed.

|  | Content  (g kg^-1^) |
| --- | --- |
| Ingredient composition  Wheat  Corn  Soybean meal, 47% CP  Soybean oil  Dicalcium phosphate  Calcium carbonate  Sodium chloride  Vitamin-mineral premix^1^  DL-methionine  L-lysine HCl  L-threonine  Ag-kaolin^2^ | 407  154  357  40  17.1  9.6  3.7  4.0  2.7  2.0  0.6  2.0 |
| Analysed composition  Dry matter  Organic matter  Crude protein  Ether extract  Neutral detergent fibre  Starch | 895  934  246  57  96  335 |
| Estimated composition  AMEn, kcal/kg  Calcium  Digestible Phosphorous | 2970  10.0  4.8 |
| Digestible Lysine  Dig. Methionine  Dig. Threonine  Dig. Tryptophan  Dig. Isoleucine  Dig. Valine | 12.0  5.6  7.6  2.5  8.5  9.4 |

^1^  Included per kilogram of diet: vitamin A, 7,520 IU; vitamin D_3_, 4,000 IU; vitamin E, 15 IU; vitamin K_3_, 2 mg; vitamin B2, 5.5 mg; vitamin B6, 2 mg; vitamin B12, 13 mcg; Zn, 52 mg; Cu, 6.2 mg; K, 1.2 mg; Se, 0.3 mg; Fe, 20 mg; niacin, 25 mg; Ca-pantothenate, 9 mg; pantothenic acid, 8 mg; folic acid, 0.5 mg; biotin, 0.1 mg; choline chloride, 100 mg; betaine, 150 mg; butylated hydroxyanisole, 0.4 mg; butylated hydroxytoluene, 2 mg; 6-phytase, 1,000 U; endo-1,4-beta-xylanase, 1220 U; endo-1,3 (4) beta-glucanase, 152 U.

^2^ Not included in control feed

**Table S4**. Composition of the gastrointestinal fluids of the pig *in vitro* digestion.

| **Constituent** | **Concentration**  **(g L^-1^)** | **Simulated salivary fluid**  **(SSF)**  **(mL)** | **Simulated gastric fluid (SGF)**  **(mL)** | **Simulated intestinal fluid (SIF)**  **(mL)** |
| --- | --- | --- | --- | --- |
| Sample |  | 0.5 g |  |  |
| SSF/SGF/SIF |  | 3.5 | 7.5 | 11 |
| KCl  KH_2_PO_4_  NaHCO_3_  MgCl_2_·6H_2_O  NaCl  (NH_4_)_2_CO_3_ | 37.5  68  84  30.5  117  48 | 15.1  3.7  6.8  0.5  -  0.06 | 6.9  0.9  12.5  0.4  11.8  0.5 | 6.8  0.8  42.5  1.1  9.6  - |
| CaCl_2_·2H_2_O |  | 0.025 | 0.005 | 0.04 |
| Ultrapure water |  | 0.975 | 0.695 | 1.31 |
| Porcine pepsin | 25000 U mL^-1^ |  | 1.6 |  |
| Bile | 160 mM |  |  | 2.5 |
| Pancreatin | 800 U mL^-1^ |  |  | 5 |
| HCl |  |  | 0.2 |  |
| NaOH |  |  |  | 0.15 |

**Table S5.** Composition of the gastrointestinal fluids of the chicken *in vitro* digestion.

| Simulated gastric fluid | Simulated intestinal fluid |
| --- | --- |
| 25 mL of 0.1 M PBS  10 mL of 0.2 M HCl  1 mL of porcine pepsin | 10 mL of 0.2 M PBS  5 mL of 0.6 M NaOH  1 mL of porcine pancreatin |

**Fig. S1.** FESEM images of the Ag-kaolin additive.


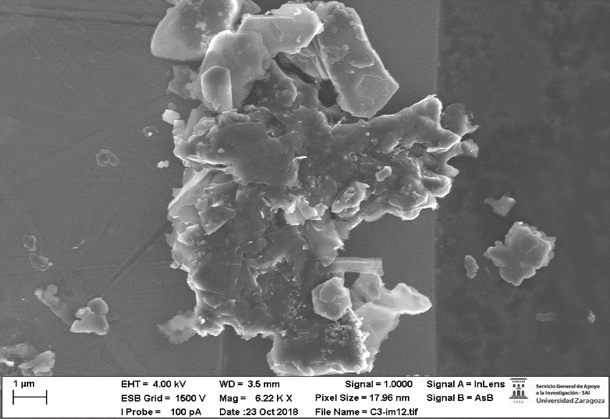


1 µm


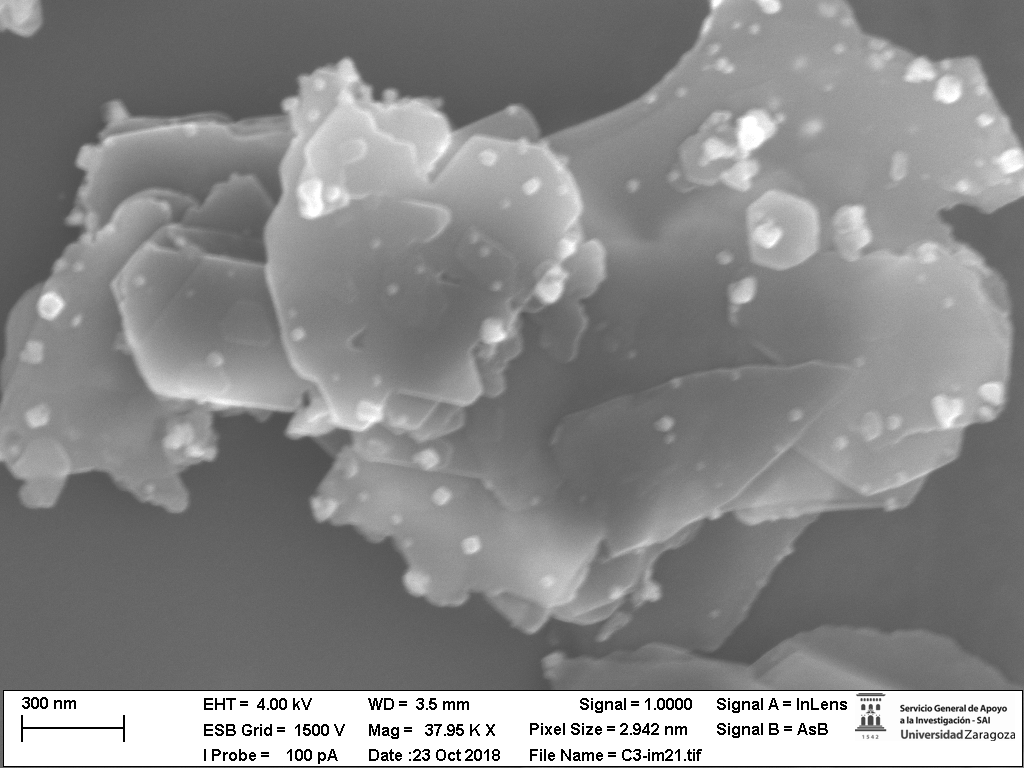


300 nm

**Table S6**. Properties of the Ag-kaolin additive.

| parameter | value |
| --- | --- |
| Ag content | 8.32 ± 0.35 mg g^-1^ |
| AgNPs mean size | 27 nm |
| pH ^(*)^ | 5.78 ± 0.16 |
| dissolution/degradation rate ^(*)^ | 2.69 ± 0.18 mg L^-1^ h^-1^ for Ag |
| zeta potential ^(*)^ | -36.3 ± 0.5 mV |

^(*)^ 1 g additive L^-1^ in ultrapure water.

**Fig. S2.** Release of total silver from Ag-kaolin in ultrapure water as a function of time (mean ± standard deviation, n=3).

**Fig. S3.** HDC-ICP-MS chromatograms. Supernatant suspension of Ag-kaolin material (1:2 dilution). Concentration: 1 g L^-1^.

**Fig. S4.** Size distribution of the supernatant of the suspension of Ag-kaolin material (1:15000 dilution). Concentration: 1 g L^-1^.

**Table S7**. Total and dissolved silver released from 1 g L^-1^ of Ag-kaolin in water (mean ± standard deviation, n=3).

| Technique | Total silver released vs. total silver  (%) | Ag(I) vs. total silver released  (%) |
| --- | --- | --- |
| Ultrafiltration + FAAS | 35.4 ± 0.8 | 99.0 ± 7.7 |
| SP-ICP-MS | 35.9 ± 2.7 | 99.9 ± 0.1 |
| HDC-ICP-MS | 33.7 ± 1.3 | 100 |

**Fig. S5**. Chromatogram of 20 nm AgNPs (red) and control feed spiked with 20 nm AgNPs (black) for the oral and gastric steps of pigs *in vitro* digestion of: (a) SSF (b) SGF

**Table S8**. Total silver concentration in pig and chicken control and supplemented feeds (mean ± standard deviation, n=10).

| Feed | | Total silver  (mg kg^-1^) |
| --- | --- | --- |
| Pig feed | Control | < LOQ |
|  | Supplemented | 12.6 ± 1.1 |
| Chicken feed | Control | 0.04 ± 0.01 |
|  | Supplemented | 12.3 ± 1.4 |

LOQ=0.012 mg kg^-1^
